# Control of Inflammation by Calorie Restriction Mimetics: On the Crossroad of Autophagy and Mitochondria

**DOI:** 10.3390/cells9010082

**Published:** 2019-12-28

**Authors:** Enrique Gabandé-Rodríguez, Manuel M. Gómez de las Heras, María Mittelbrunn

**Affiliations:** 1 Immunometabolism and Inflammation Laboratory, Tissue and Organ homeostasis Program, Cell-Cell Communication and Inflammation Unit, Centro de Biología Molecular Severo Ochoa (CBMSO), Consejo Superior de Investigaciones Científicas-Universidad Autónoma de Madrid (UAM), 28049 Madrid, Spain; manuel.montero@cbm.csic.es; 2 Department of Molecular Biology, Faculty of Sciences, Universidad Autónoma de Madrid (UAM), 28049 Madrid, Spain; 3 Regulation of Cellular Homeostasis Laboratory, Area of Rare and Genetically Based Diseases, Instituto de Investigación Hospital 12 de Octubre, 28041 Madrid, Spain

**Keywords:** autophagy, mitochondria, calorie restriction, aging, metabolism, inflammation

## Abstract

Mitochondrial metabolism and autophagy are two of the most metabolically active cellular processes, playing a crucial role in regulating organism longevity. In fact, both mitochondrial dysfunction or autophagy decline compromise cellular homeostasis and induce inflammation. Calorie restriction (CR) is the oldest strategy known to promote healthspan, and a plethora of CR mimetics have been used to emulate its beneficial effects. Herein, we discuss how CR and CR mimetics, by modulating mitochondrial metabolism or autophagic flux, prevent inflammatory processes, protect the intestinal barrier function, and dampen both inflammaging and neuroinflammation. We outline the effects of some compounds classically known as modulators of autophagy and mitochondrial function, such as NAD^+^ precursors, metformin, spermidine, rapamycin, and resveratrol, on the control of the inflammatory cascade and how these anti-inflammatory properties could be involved in their ability to increase resilience to age-associated diseases.

## 1. Introduction

Inflammation is a protective response that is triggered under certain threatening conditions promoting the elimination of damage, tissue repair, and the recovery of homeostasis. An acute inflammatory response is typically activated upon infection or tissue injury and involves the recruitment of immune cells to the site of damage. The coordination of immune cells mobilized from the bloodstream, in conjunction with tissue resident immune cells, ensures the elimination of the damage followed by the resolution of the inflammatory process. Both the innate immune system, that quickly responds to the initial damage, and the adaptive immune system, that confers immunological memory and promotes faster responses to repeated infections, are essential to coordinate a successful inflammatory response. Under certain circumstances such as aging, there is a failure in the resolution mechanisms leading to the chronic activation of immune cells and persistent inflammation. This state of low-grade but chronic inflammation is known as inflammaging, and is characterized by increased levels of pro-inflammatory cytokines in the circulation [1,2]. Notably, inflammaging is considered a risk factor for many age-related diseases [3,4]. Even in certain tissues like the brain, that possesses a privilege protection against inflammation, certain signs of inflammation appear gradually with age, and this neuroinflammation can anticipate the appearance of some neurodegenerative diseases [5,6,7]. In addition, the integrity of the intestinal barrier is compromised due to inflammatory stress during aging and contributes to the development of several diseases [3,8]. Finding drugs that protect against inflammaging, the disruption of the intestinal barrier, and neuroinflammation should be a priority for geroscience in the next years.

**Figure 1:**
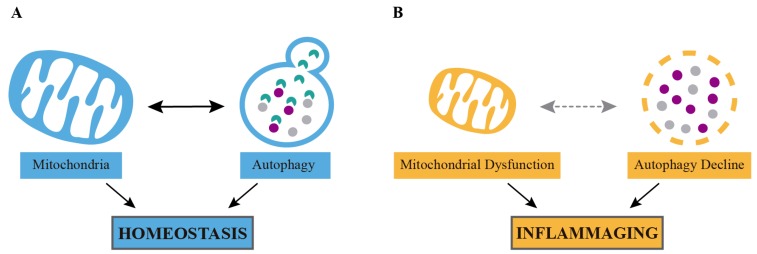
Mitochondria and autophagy interplay during inflammation and aging. (**A**) Mitochondria metabolism and autophagy are two of the most metabolically active cellular pathways, both plying critical roles in the regulation of the metabolic health and the organism homeostasis. (**B**) However, the impairment of the mitochondrial function, the autophagy pathway, or its crosstalk lead to inflammation and aging.

Mitochondrial metabolism and autophagy are two of the most metabolically active cellular processes, playing a crucial role in regulating organism longevity. It is well known that an intense crosstalk exists between mitochondria and autophagosomes [9], and the activity or stress status of either one of these organelles may affect the other (Figure 1). A mitochondrial or autophagy decline compromises cellular homeostasis and induces inflammation. Furthermore, mitochondrial function and autophagy are key pathways controlling the activation of both the innate and the adaptive immune system [10,11].

Mitochondria control a plethora of processes in the cell [12], not only by controlling ATP production, but also by serving as biosynthetic and signaling centers. In fact, in the last decade, it has become evident that mitochondria are essential organelles that direct the fate of immune cells, giving rise to a new scientific discipline that is called Immunometabolism. Moreover, the outcome of the inflammatory response can be controlled by modulating the metabolism of immune cells [13]. 

Macroautophagy, here called autophagy, is a cellular process responsible for the degradation of protein aggregates and damaged organelles. During autophagy, double membrane vesicles, named autophagosomes, engulf and transport cargo, which is subsequently degraded following the fusion of the autophagosomes with lysosomes to form autophagolysosomes. Autophagy degrades several organelles, highlighting the degradation of mitochondria through autophagy degradation, which is known as mitophagy. Mitophagy plays an important role in the control of cellular homeostasis [14]. The molecular mechanisms controlling mitophagy have been well characterized in recent years. In unhealthy mitochondria, PINK1 acts as a sensor of mitochondrial quality by accumulating in the mitochondrial membrane and recruiting the E3 ubiquitin ligase Parkin. Parkin ubiquitinates different substrates of the mitochondrial membrane, allowing the recognition of the mitochondria by adaptor proteins that bind LC3-II in the autophagosome membrane. The correct recognition of the damaged mitochondria facilitates efficient engulfment and degradation [14]. 

Remarkably, both mitochondria and autophagy function dramatically impact the aging process (Figure 1). Harman’s mitochondrial theory of aging proposed that accumulation of mitochondrial DNA (mtDNA) mutations impairs mitochondrial respiration and leads to the accumulation of mitochondrial reactive oxygen species (mtROS). This accelerates the appearance of new mtDNA mutations, resulting in a vicious cycle that would cause cellular aging [15]. Indeed, mutations affecting the stability, transcription, or translation of the mtDNA are known to induce progeroid-like phenotypes by affecting the stability of the mitochondrial pool [16]. In addition, autophagy induction has been constantly proposed as a way to fight aging symptoms. Thus, mice with the tissue specific deletion of genes that control autophagy or mice with a whole body downregulation of autophagy present degenerative signs resembling those associated with aging and age-associated diseases, such as neurodegeneration [17,18], Alzheimer’s disease (AD) [19], increased lipid storage [20], muscle atrophy [21], hyperglycemia [22], and cardiac [23] or renal dysfunction [24]. Importantly, autophagy is downregulated during aging in worms [25,26], *Drosophila* [27], and mammals [26], and its induction by pharmacological or genetic approaches promotes longevity and healthspan in different experimental models [28,29,30,31,32].

Recent findings exemplify the clear interconnection that exists between both mitochondria and autophagy. Importantly, an impairment of this crosstalk favors the activation of several inflammatory pathways, both in pathological conditions and during aging. For instance, inhibition of autophagy promotes the accumulation of dysfunctional mitochondria facilitating the release of mtDNA to the cytoplasm. Cytosolic mtDNA increases Caspase-1 activation and IL-1β production, exemplifying the importance of the interconnection of both mitochondria and autophagy in the control of IL-1β production [33,34,35]. Further examples have shown that autophagy modulates the activation of the inflammatory transcription factor NF-κB [36]. Similarly, mitochondrial dysfunction and the consequent rewiring of the metabolism towards glycolysis favors the acquisition of a pro-inflammatory phenotype in different immune cells [13,37]. Besides its role in controlling cellular energetics and metabolism, mitochondria are considered signaling hubs [12] that modulate intracellular pools of Ca^2+^ and ROS. These molecules are classical mediators of inflammation [38,39]. Regarding Ca^2+^, mitochondria regulate both longevity and the fate of the inflammatory response through tethering to other organelles such as the ER. For instance, the maintenance of mitochondria-ER contact sites regulates leukocyte migration and lymphocyte activation by balancing the Ca^2+^ intracellular pools and by regulating autophagy induction [40]. Concerning the increase in ROS, cumulative evidence has led to formulate the oxidation-inflammation theory of aging that postulates a vicious circle in which the increased production of ROS and inflammatory mediators, known as oxi-inflamm-aging, promote a further production of both noxious molecules during aging [41].

**Figure 2:**
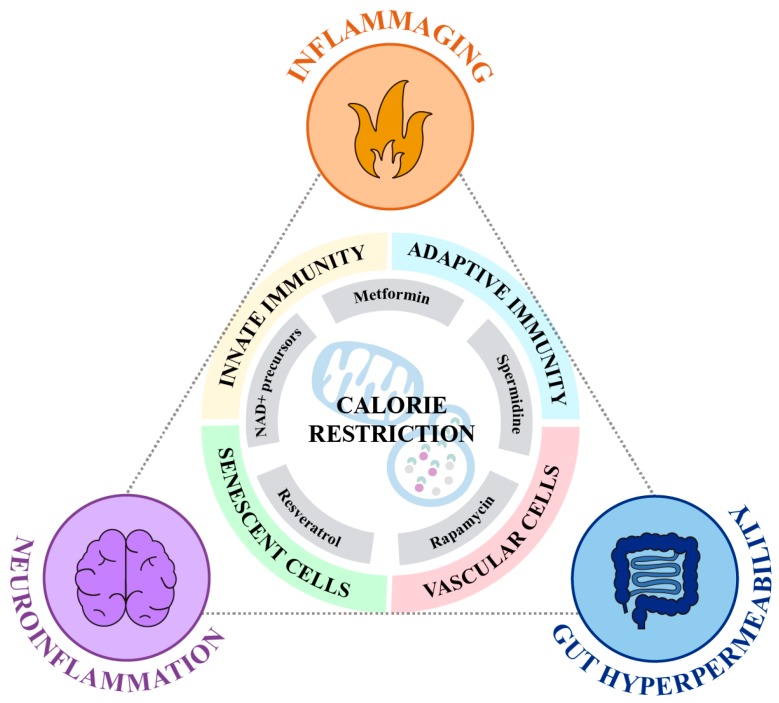
Calorie restriction and CR mimetics modulate inflammaging, neuroinflammation and gut permeability. Calorie restriction and its mimetics act on autophagy and mitochondrial function to prevent the activation of inflammatory pathways. Its anti-inflammatory role occurs both at the systemic (inflammaging) and at the local level (i.e., neuroinflammation, and enhanced gut barrier permeability).

Due to the clear importance of autophagy and mitochondria in conferring resilience to age-related diseases, the potential anti-aging effects of interventions which induce the activation of any, or both pathways, has been explored in detail. One of the first anti-aging approaches to attract attention in the field was calorie restriction (CR). As illustrated below, CR induces autophagosome formation, mitophagy and rises cellular levels of nicotinamide adenine dinucleotide (NAD^+^), improving autophagy function and mitochondrial fitness. Interestingly, CR protocols have shown potent anti-inflammatory properties [42]. In light of the findings regarding the beneficial effects of CR diets, several efforts have been made in generating and characterizing new compounds, known as CR mimetics, that mimic the effects of CR without the evident unpleasant consequences of food intake restriction (Figure 2). However, the role played by the downregulation of inflammation as a potential mechanism mediating the effect of these compounds has been consistently underrated.

Herein, we outline the effects of compounds classically known as modulators of autophagy and mitochondrial function on the control of the inflammatory cascade (Table 1). These anti-inflammatory effects, which remain poorly defined, could mitigate inflammaging, intestinal barrier disruption, and neuroinflammation, improving resilience to aging and to the appearance of age-related diseases.

## 2. Anti-Inflammatory Effects of Calorie Restriction 

CR is probably the oldest strategy known to promote healthspan. Actually, first evidences dated from AD 1000, when the Persian polymath Avicenna already taught the elderly to eat less than when they were young [43]. More recent research on aging has focused on unraveling the molecular mechanism by which CR acts. Several reports have characterized that the effects of CR on lifespan extension are mediated by the activation of Sirtuin-1 (Sirt1) [44] and autophagy [45,46]. In addition, CR reduces adiposity and alters Insulin-like Growth factor 1 (IGF-1) signaling. Thus, insulin receptor knockout in the adipose tissue extends longevity in mice [47]. Indeed, insulin downstream signaling inhibits the Forkhead Box proteins (FOXO). Therefore, PHA-4, the *C. elegans* orthologue of the human FoxA transcription factors, is required for CR-induced longevity [48]. In addition to autophagy promotion, CR promotes mitochondrial biogenesis in humans [49] and corrects the expression of several genes affected by aging, whose function is related to mitochondrial biogenesis and function [50]. CR also exerts anti-aging effects by reducing oxidative stress through a Sirt3-dependent activation of superoxide dismutase 2 [51].

One of the mechanisms that has emerged as a potential candidate of CR action is the downregulation of inflammatory pathways. Indeed, CR is able to decrease inflammation in several experimental models. For example, CR normalizes TNF-α and IL-6 serum levels in old mice up to young mice levels [52], and promotes a youthful transcriptional profile that includes downregulation of inflammatory pathways in rats and middle age humans [53]. Likewise, β-hydroxybutyrate, a ketone metabolite that accumulates during CR, mediates an anti-inflammatory effect by blocking the NLRP3 inflammasome and the subsequent IL-1β/IL-18 production in human monocytes and mouse models of different inflammatory diseases [54]. Recently, a very elegant study has shown that fasting increases AMP-activated protein kinase levels (AMPK), modulates the metabolic activity of monocytes, including a decrease in OXPHOS, and reduces the steady state levels of CCL2, thus precluding monocytes from leaving the bone marrow (BM). Of note, by doing so, fasting improves the phenotype of mice undergoing experimental autoimmune encephalomyelitis (EAE) without altering the immune response to bacterial infections [42]. 

CR has also shown important immunomodulatory effects on adaptive immune cells. Upon CR, high levels of glucocorticoids (GC) in the circulation favor the reorganization of the BM and the recruitment of memory T cells into it, in an attempt to avoid high levels of circulating GC. Once in the BM, the memory T cells activate the expression of pro-survival factors such as Bcl-2 or mTOR, which license them to remain longer in the BM stroma. Upon a second infection, in CR conditions, the increased reservoir of memory T cells that survive in the BM is once again mobilized into the bloodstream, improving fighting against infection [55]. Considering the decrease in the efficiency of the immune response during aging [56], CR could improve resistance to infection in old organisms by this mechanism. Additionally, a recent report has shed light on the mechanisms by which autophagy and caloric restriction could confer potent anti-tumor activity to T cells. The authors found that high levels of K^+^ in the extracellular space of tumors activate a functional CR molecular program in surrounding effector T cells. This activation promotes autophagy activation and metabolic reprograming of CD8^+^ T cells, encouraging mitochondrial respiration and activation of the TCA [57].

CR has also demonstrated some important anti-inflammatory properties in the context of neurological diseases. CR attenuates microglia activation in lipopolysaccharide (LPS)-injected mice [58] and in cortical injury rat models [59]. Furthermore, CR ameliorates behavioral deficits in an AD mouse model [60]. CR also improved behavioral and molecular signs in a MPTP-induced mouse model of Parkinson’s Disease (PD) by increasing the levels of the brain derived neurotrophic factor and the glial cell-derived neurotrophic factor [61]. The anti-neuroinflammatory properties of CR have also been extrapolated to EAE models where it decreases IFN-γ and IL-6 levels and ameliorates demyelination [62].

The effect of CR on preserving the integrity of the intestinal barrier has been tested in rats and humans. In rats, long-life CR does not reverse the loss of permeability observed in old animals [63]. In contrast, a mild four-week of CR improves systemic inflammation and the permeability of the intestinal barrier in obese humans [64]. 

## 3. Calorie Restriction Mimetics

### 3.1. NAD^+^ Precursors

NAD^+^ synthesis is required for the effects of CR on lifespan extension [65]. The molecular mechanisms underlying NAD^+^ precursor effects have been largely attributed to the activation of Sirt1, an important molecule that modulates the crosstalk between autophagy and mitochondrial function. Thus, NAD^+^ levels decline during aging, disrupting mitochondrial respiration through the impaired transcription of nuclear encoded mitochondrial subunits [66]. Moreover, NAD^+^ activates Sir2p, the yeast homolog of the mammalian Sirt1, which de-acetylates Ku70, a DNA repair factor that sequesters Bax away from mitochondria, thereby inhibiting apoptosis and inducing lifespan extension [67]. In humans, SNP variants of the *FOXO3* gene, a well-known regulator of autophagy [68,69,70,71], are associated with longevity in centenarians [72]. Interestingly, Sirt1 modulates the levels of the Forkhead Box O3A (FOXO3A) [73,74]. Accordingly, NAD^+^ precursor supplementation induces mitophagy and improves healthspan and lifespan in *Drosophila*, *C. elegans* [75] and in mouse models of accelerated aging [76]. Thus, NAD^+^ has emerged as a potent anti-aging factor, and the use of NAD^+^ precursors has been proposed as a potent strategy to target age-associated diseases in old organisms and invertebrate models of premature aging syndromes [75]. 

Importantly, NAD^+^ precursor compounds are potent anti-inflammatory drugs [77]. Nicotinamide mononucleotide (NMN) supplementation, a product of the nicotinamide phosphoribosyltransferase (NAMPT), which acts as the rate limiting enzyme in the NAD^+^ biosynthesis, decreases the levels of inflammatory cytokines in mice fed on a high fat diet (HFD) and in a mouse model of aged-induced type 2 diabetes. This effect seems to be mediated by a Sirt1 dependent de-acetylation of NF-κB and, remarkably, correlates with improved glucose and lipid homeostasis [78]. NMN treatment also decreases IL-1β production in mouse models of diabetes [79] and in the muscle of old mice [66]. Additionally, NAM is also effective in various inflammatory skin conditions [80]. Intriguingly, Van Gool et al. reported that, in LPS-stimulated macrophages, inhibition of NAMPT blunts the production of TNF-α, but not IL-6 or IL-1β, in a Sirt6-dependent manner [81]. This exemplifies the complexity of the relationship between NAD^+^ metabolism and the regulation of the different inflammatory cascades.

The anti-inflammatory effect of NAD^+^ boosting compounds is also extended to diseases affecting the nervous system [82]. Nicotinamide riboside (NR) supplementation reduces microgliosis, astrogliosis and cytokine secretion in a mouse model of AD [83]. Interestingly, NAD^+^ supplementation activates mitophagy and inhibits the NLRP3 inflammasome [84] which is hyperactivated in disease [85,86,87]. In addition, during AD progression, microglia phagocytose A thus protecting from its toxic effects [88]. Similarly, NR treatment induces mitophagy, DNA repair and improves lifespan and healthspan in mouse models of ataxia telangiectasia [88]. Interestingly, NAD^+^ decline during aging, due to reduced de novo synthesis, impairs phagocytosis and the resolution of inflammation in macrophages, suggesting this could be another potential mechanism by which NAD^+^ precursors reduce neuroinflammation in AD models [89]. Likewise, supplementation with NMN decreased microglia activation, neutrophil infiltration and TNF-α and IL-6 levels in mouse models of intracerebral hemorrhage [90]. NAD^+^ supplementation also efficiently worked in mouse models of EAE, in which it blocks disease progression by regulating T cell differentiation through the activation of the tryptophan hydroxylase-1 (Tph1). The activation of Tph1 skews T cell differentiation towards CD4^+^IFNγ^+^IL-10^+^ subsets, favoring immune homeostasis and preventing demyelination [91]. Similarly, NAM reduces the infiltration of immune cells and the demyelination in EAE models [92].

Recently, the anti-inflammatory properties of NAD^+^-boosting compounds have been extended to the human scenario. The short-term administration (21 days) of NR to aged individuals increases NAD^+^ levels in the muscle and reduces the expression of the inflammaging-associated cytokines IL-6, IL-5, and IL-2 [93]. This study opens a door for longer NR interventions in aged humans. 

### 3.2. Resveratrol

Resveratrol is a natural phenol produced by several plants in response to injury or infections. Natural sources of resveratrol include grapes, blueberries or peanuts. Pioneer studies showed that resveratrol is able to extend longevity in *Saccharomyces cerevisiae* and *Drosophila* [44,94]. In mice, resveratrol administration improves healthspan without extending longevity [95]. However, in mice under a high-caloric diet, resveratrol extends both healthspan and lifespan [96] and improves insulin sensitivity [97]. Moreover, in humans, short term administration (30 days) to obese individuals improves several metabolic parameters, probably by promoting mitochondrial function in the muscle [98].

Regarding the molecular mechanism, resveratrol is considered a CR mimetic that works by activating Sirt1. Park et al. showed that resveratrol increases the intracellular levels of cAMP, resulting in an increase in the intracellular levels of Ca^2+^. This activates the CamKK-AMPK pathway, via phospholipase C, and the ryanodine receptor Ca^2+^-release channel which increases NAD^+^ levels and Sirt1 activation [99]. Notably, Sirt1 dependent activation of autophagy mediates longevity extension in *C. elegans* [100]. Furthermore, resveratrol can also impact on mitochondrial function by inducing mitophagy and by modulating mitochondrial dynamics both in vitro [101,102] and in vivo [103,104,105]. In addition to Sirt1 activation, resveratrol works as a scavenger of different ROS, thus protecting from oxidative stress [106]. 

Cumulative studies have demonstrated the anti-inflammatory properties of resveratrol [107]. In old mice, resveratrol treatment reverses the increase in the secretion of the pro-inflammatory cytokines TNF-α, IL-6, IL-1β as well as the levels of ICAM-1 and the inducible nitric oxide synthase (iNOS) [95]. In addition, it decreases inflammation in the adipose tissue of rhesus monkeys under high fat and sugar diets [108]. Similarly, resveratrol treatment downregulates the production of some circulating inflammatory markers such as IL-1β, TNF-α, IL-6 and IL-8, conceivably, by decreasing the number of circulating leukocytes in obese humans [98]. Coinciding with the importance of Sirt1 in the regulation of macrophage activation [109], strong evidence supports the anti-inflammatory effects of resveratrol, in part, due to its action on innate immune cells [109]. Resveratrol attenuates the activation of the NF-κB pathway in vitro [110], suggesting that the decrease in circulating cytokines and inflammatory markers is not an indirect, beneficial effect derived from its effect on non-immune cells. Actually, resveratrol inhibits NF-κB and Nitric Oxide production in macrophages in vitro [111]. Additionally, resveratrol could regulate the activation of important inflammatory pathways, such as the NF-kB pathway, by scavenging ROS [107]. 

The effect of resveratrol on adaptive immune cells has been tested in different models of T cell-dependent inflammation, like EAE, in which it effectively decreases inflammation. It was shown that at least some of the effects of resveratrol on EAE could be mediated by an increase in the number of IL-17^+^ IL-10^+^ T cells [112]. However, other authors have stated that this effect could rather be due to an induction of T cell apoptosis via activation of the aryl hydrocarbon receptor and a concomitant decrease in the number of T cells [113], or due to decreased T cell proliferation [114]. 

The anti-inflammatory properties of resveratrol go beyond its action on immune cells. For instance, resveratrol modulates the adhesion of leukocytes to endothelial cells, a crucial step in the inflammatory cascade [115]. Thus, resveratrol attenuates inflammation in human umbilical vein endothelial cells (HUVEC) cells by a mechanism that depends on the Sirt1 mediated activation of autophagy [116]. Resveratrol also decreases the levels of important adhesion molecules, such as ICAM-1, in TNF-α stimulated endothelial progenitor cells [117] and HUVEC cells [118]. 

The beneficial effects of resveratrol extend to neuroinflammation. Sirt1 activation protects microglia cells from activation upon Aβ exposure. Thus, in those conditions, resveratrol promotes microglia quiescence by inhibiting NF-κB activation and the subsequent production of pro-inflammatory cytokines, protecting neurons from dying in co-culture experiments [119,120]. In vivo, resveratrol ameliorates disease signs and astrocytosis in the p25 transgenic model of AD and in cellular models of amyotrophic lateral sclerosis [121].

### 3.3. Metformin 

Metformin is a drug derived from galegine, a natural product from the plant *Galega officinalis* used as a standard treatment for diabetes. While the exact mechanism by which metformin improves diabetes is not fully understood, cumulative evidences show that this compound can target a wide range of molecular pathways related to aging and therefore, it has been postulated as a potential treatment for aging-associated diseases. One of the preferential targets that is activated by metformin is AMPK [122]. As mentioned, AMPK regulates autophagy [123] and mitochondrial dynamics, and is also activated by CR, thus mediating at least some of the beneficial effects that CR has on healthspan and lifespan extension [124]. Accordingly, metformin extends lifespan and healthspan in *C. elegans* and mice [125]. While AMPK activation is the most characterized effect of metformin, several reports have shown that it also inhibits the mitochondrial complex I, which is essential during the OXPHOS [126]. This leads to a decrease in mtROS production that can be protective in certain situations [127].

Because AMPK and mitochondrial respiration are two of the multiple potential pathways that metformin can target, it has been postulated that autophagy and mitochondrial function might be mediators of its beneficial effects on lifespan and healthspan extension [125]. Interestingly, metformin is a potent anti-inflammatory drug and, thus, it has been postulated that some of the effects of metformin on healthspan and lifespan extension could be mediated by the control of the immune system response [128]. Some evidence from in vitro experiments have shown that metformin is able to diminish the expression of adhesion molecules such as E-selectin, ICAM-1, VCAM-1 in TNF-α activated endothelial cells. The underlying mechanism would be mediated by an inhibition of the NF-κB pathway [129]. The vascular NF-κB-dependent anti-inflammatory effect of metformin has been further confirmed in IL-1β stimulated endothelial cells and vascular smooth muscle cells [130]. Strikingly, by targeting the NF-κB pathway, metformin also inhibits the pro-inflammatory senescence associated secretory phenotype (SASP) of senescent cells [131].

Importantly, several evidences suggest that metformin can also act as an anti-inflammatory drug in vivo. Old mice treated with metformin show decreased activation of the pro-inflammatory pathways NF-κB and JNK and increased levels of the anti-inflammatory cytokine IL-10 [132]. In addition, mice presenting non-alcoholic fatty liver disease (NAFLD), a disease that is clearly associated with aging, present an improved profile of inflammatory markers following metformin administration [133]. An additional potential mechanism could be mediated by a Sirt1-dependent activation of the autophagy pathway [134]. 

Another possible anti-inflammatory effect of metformin arises from the modulation of lymphocyte differentiation. During aging, an increased number of antigen-naïve but semi-differentiated memory T cells, known as virtual memory T cells, accumulate and impair proper response to antigens [135]. Strikingly, metformin promotes CD8^+^ memory T cell differentiation, by activating a TNF receptor associated factor 6 (TRAF-6)-dependent induction of fatty acid metabolism [136]. This suggests that improved adaptive immune function could mediate some of the anti-inflammatory effects that metformin has demonstrated during aging. 

Interestingly, metformin has also been proven to be effective in combating neurological diseases associated with inflammation. Specifically, metformin restricts the entry of mononuclear cells into the central nervous system of a mouse model of EAE, downregulating the expression of several inflammatory markers and cytokines like TNF-α, IFN-γ, IL-6 or IL-17 [137]. Similar efforts have been made using metformin for the treatment of AD, which is the most common age associated neurodegenerative disease worldwide. However, while the wide range of targets that metformin possess could be on one hand beneficial for treating different age associated diseases, the potential of metformin on targeting AD is still very controversial. Thus, while it increases the production of the AD-associated neurotoxic peptide A, by upregulation of the β-site APP cleaving enzyme 1 (BACE-1) activity [138], it results protective in Aβ-driven models of AD, in which it efficiently decreases microglia and astrocyte activation and NF-κB signaling [139]. Moreover, it reduces the phosphorylation of Tau protein, the other neurotoxic protein accumulated in AD, by activating the phosphatase PP2A [140] and is able to induce the autophagy mediated clearance of toxic aggregates [141]. Therefore, the use of metformin in AD patients, even though it could have potential neuroinflammatory properties, should be taken with caution. 

PD is a neurodegenerative disease affecting the dopaminergic neurons of the substantia nigra, thus causing motor coordination impairment and late onset dementia [142]. Mutations in the mitophagy associated genes *PINK1* and *PARKIN* are responsible of early onset PD [143,144]. Interestingly, recent evidence has shown that *Parkin* and *Pink1* deficient mice develop activation of the stimulator of interferon genes (STING) and inflammation which is directly responsible for the death of dopaminergic neurons in a PD mouse model [145]. Metformin supplementation ameliorates the signs of stroke, cytokine levels, and neutrophil infiltration in the brains of mice subjected to medial cerebral artery occlusion by activating the AMPK pathway and inhibiting NF-κB signaling [146,147]. In contrast, in an inflammatory model of PD consisting of intranigral injection of LPS, metformin ameliorates microglial activation and cytokine secretion, but exacerbates the death of dopaminergic neurons [148]. These detrimental effects may arise from the fact that metformin is a weak inhibitor of the mitochondrial complex I [149], whose deficiency has been clearly linked to PD development [150]. In addition to brain diseases, a recent report has demonstrated that metformin can improve remyelination in the spinal cord of old rats by enhancing mitochondrial function in oligodendrocytes through the activation of AMPK [151]. Importantly, a chronic activation of the inflammasome, which is known to be inhibited by metformin [152], impairs remyelination during aging [153], suggesting that some of the beneficial effects of metformin in remyelination models could work by dampening age-associated inflammation.

Some studies have also addressed the potential of metformin on precluding intestinal barrier disruption. Metformin treatment in IL-10 knockout mice, that display intestinal inflammation [154], increased AMPK signaling, decreased the number of pro-inflammatory M1 macrophages and the levels of TNF-α, IL-1β and IFN-γ [155]. Similar effects were found in a mouse model of dextran sodium sulphate (DSS)-induced intestinal inflammation [156]. However, the effects of metformin treatment in the age-associated loss of permeability of the intestinal barrier still remains to be addressed. 

### 3.4. Spermidine

Spermidine is a natural polyamine, usually administered as a dietary compound, that extends longevity in yeast, worms, and flies by inducing autophagy [157,158]. Interestingly, spermidine is also able to improve mitochondrial function, possibly by increasing the degradation of damaged mitochondria through mitophagy [159]. These intriguing properties have led to several studies demonstrating the protective effect of spermidine treatment on different age-associated diseases. Spermidine administration demonstrated to be cardioprotective in old mice, reducing cardiac hypertrophy and improving diastolic function and, most importantly, spermidine intake negatively correlates with cardiovascular risk in humans. Of note, spermidine reduces the levels of the inflammaging-associated cytokines IFN-γ, IL-1β, IL-6, and TNF-α in mice [159]. 

An important step in the inflammatory cascade involves the adhesion of immune cell molecules to the endothelium and the directional migration of these cells towards the injured tissue [160]. One of the key molecules mediating this process is the adhesion molecule LFA-1 [161]. Notably, the transcription of *Itgal*, the gene encoding for LFA-1, is increased in blood cells from old human donors [162]. Interestingly, spermidine induced hypermethylation of the *Itgal* gene and suppressed LFA-1 expression in lymphocytes [162], suggesting that another anti-inflammatory effect of spermidine could involve epigenetic inhibition of leukocyte migration.

The direct effects of spermidine have also been well characterized in immune cells. In aged humans, T cell function declines with age alongside with a decrease in autophagy [163]. In old mice, impaired CD8^+^ T cell response against influenza vaccination is restored by spermidine in an autophagy dependent manner. The induction of autophagy is however independent of mTORC1 activation, suggesting that spermidine and rapamycin act through different pathways in T cells [164]. Spermidine blocks the production of the pro-inflammatory cytokines IL-12 and IFN-γ and enhances the production of the anti-inflammatory cytokine IL-10 in LPS-stimulated mouse macrophages [165]. The anti-inflammatory effects of spermidine on macrophages were further validated in LPS-stimulated human peripheral blood mononuclear cells, in which it inhibits the production of TNF-α, IL-1, MIP-1a and MIP-1b and in mouse models of carrageenan-induced footpad inflammation [166]. Another age-associated feature that is very sensitive to increased inflammation is the maintenance of the intestinal barrier, which controls the entry of pathogens and toxic compounds into the circulation as well as the absorption of ingested nutrients [167]. Intriguingly, spermidine protects epithelial cells from the inflammatory effects of IFN-γ in vitro, and is able to restore transepithelial electrical resistance through the activation of the T cell protein-tyrosine phosphatase (TCPTP) [168].

Spermidine also has direct in vitro anti-inflammatory properties in microglia, at least when stimulated by LPS through a mechanism that involves the downregulation of NF-κB activation [169]. Furthermore, spermidine rescues age-associated loss of memory in flies by promoting autophagy [170] and it was found to be neuroprotective in mice undergoing EAE, a model that highly depends on immune cell activation. Surprisingly, the effect on EAE was T cell-independent, but due to skewed differentiation of macrophages towards the M2 phenotype, a blockage in the production of IL-1, IL-12, and CD80 and induced the expression of arginase-1 [171]. 

### 3.5. Rapamycin

Rapamycin, also known as sirolimus, is a macrolide compound isolated from *Streptomyces hygroscopicus* that inhibits the mammalian target of rapamycin (mTOR) complex. Overall, rapamycin has probably been the most studied anti-aging drug. This molecule extends lifespan in both male and female mice of genetically heterogeneous background [30] and in *Drosophila* [172]. Accordingly, genetic deletion of the S6K1, an mTOR downstream effector, increases mouse lifespan [173] and protects from age and diet induced obesity [174] and cardiac dysfunction [175]. While most of the anti-aging effects of rapamycin have been attributed to an induction of autophagy, which is inhibited by mTOR signaling [123,176], these effects could also be mediated by an induction of mitonuclear protein imbalance and activation of the mitochondrial Unfolded Protein Response (UPR^mt^) in *C. elegans* [177]. However, the effect of the UPR^mt^ on lifespan extension in *C. elegans* remains controversial [178]. Chin et al. found a further connection between mTOR, mitochondrial function and lifespan extension. These authors found that mTOR inactivation partially mediates the lifespan extension effects of α-ketoglutarate, an intermediate of the TCA cycle, in *C. elegans* [179]. 

Importantly, rapamycin possesses potent immunomodulatory properties and is actually used as an immunosuppressant drug in patients receiving organ transplantation [180]. The phenotype of several immune cells is determined by a balance between OXPHOS and glycolysis. Thus, while resting macrophages mainly rely on OXPHOS for energy production, pro-inflammatory activated macrophages show high glycolytic rates [181]. Moreover, mTOR signaling controls this metabolic switch by inducing the expression of HIF-1α. Accordingly, rapamycin treatment inhibits TNF-α production in β-glucan-stimulated monocytes [182,183]. Rapamycin has also been shown to be effective in inhibiting natural killer cell (NK) activation through IL-15 signaling [184]. Furthermore, chronic rapamycin treatment alters gene expression in immune cells, reduces the number of PD1^+^ T cells, which accumulate during normal aging [185], and increases the lifespan of non-infected immunocompromised mice [186]. Intriguingly, rapamycin treatment increases the number of memory precursors during the T cell expansion phase and accelerates the memory cell differentiation of CD8^+^ T cells during the effector to memory transition, thus increasing the number and quality of memory CD8^+^ T cells. This points to rapamycin as an efficient treatment to improve responses after vaccination and against viral infections [187], which are known to be impaired during aging [135].

The anti-inflammatory properties of rapamycin go beyond immune cells, as it is also able to block the production of SASP-associated cytokines of senescent cells and is effective in reducing the pro-tumorigenic activity of those cytokines [188]. In a recent, interesting study, Correia-Melo et al. tested the effects of rapamycin treatment in the *nfkb1^−/−^* mouse model of inflammaging. These mice lack p105 and p50 expression, which leads to the over activation of the FB pathway and induction of chronic low-grade inflammation. Interestingly, rapamycin treatment prevents age-related frailty without altering inflammation by decreasing the number of senescence cells [189]. 

Rapamycin has been shown to exert beneficial effects against neurological diseases, as well. Initial seminal research attributed these beneficial effects to an enhancement of the autophagy pathway, which is responsible for the degradation of several toxic proteins whose accumulation cause some neurodegenerative diseases [176,190,191]. While autophagy-mediated clearance of toxic aggregates is undoubtedly a causative reason for rapamycin’s neuroprotectiveness, targeting the inflammation associated with the accumulation of toxic aggregates may be a mediator of the effects of rapamycin. Thus, rapamycin treatment ameliorates microglia activation and disease signs in mouse models of the mitochondrial disease Leigh syndrome [192], cerebral palsy [193], traumatic brain injury [194], and tauopathy [195]. Even though these studies are based on correlative evidence, similar investigations have shed light on the mechanisms mediating the neuroinflammatory actions of rapamycin. For instance, in mouse models of EAE, mTOR activation in T cells promotes HIF-1α signaling and glycolysis. Glycolytic T cells preferentially differentiate towards pathogenic Th17 cells instead of protective regulatory T cells and accordingly, mTOR inhibition by rapamycin improves EAE signs [196]. Rapamycin inhibits the infiltration of gamma delta T cells and granulocytes in the brain of mice undergoing ischemic damage, which results in decreased number of M1 microglia and increased numbers of T reg cells, thus attenuating brain damage and motor impairment [197,198]. Dzamko et al. partially reconciled the controversy between the dual effect of rapamycin on autophagy and inflammation. They found that neuronal but not microglial increases in TLR2 signaling occur in PD patients and activate neuroinflammatory signaling pathways. As autophagy inhibition induces the accumulation of α-synuclein, rapamycin was capable of efficiently decreasing the TLR2-dependent accumulation of α-synuclein, evidencing the clear interconnection of both pathways [199]. 

Rapamycin treatment has shown some properties in preserving the age-associated loss of intestinal barrier integrity. In aged *Drosophila*, rapamycin dependent inhibition of TOR activates the FoxA transcription factor homolog Fork Head (FKH) in the gut and promotes longevity extension [200]. These effects are not mediated by altered microbiota composition but due to enhanced autophagy [32].

## 4. Concluding Remarks

In the last century, the conjunction of a decrease in birth rates and an overall extension of life expectancy have led to a progressive increase in the average age of the population worldwide, especially in first-world countries. The discovery of several molecular pathways associated to aging, and the characterization of the beneficial effects of CR, have fostered the emergence of a large number of anti-aging drugs, whose effects on lifespan and healthspan extension have been well characterized in different organisms. Most of these drugs impact mitochondria metabolism or autophagy. Here, we highlight how these drugs, by modulating these metabolic pathways, are able to prevent inflammation, and how this unappreciated role could be essential to promote lifespan and healthspan extension (Table 1). Inflammation has been considered for years a mere marker of biological aging. However, growing evidence supports the fact that inflammation is actually a causative driving-aging factor. Applying the knowledge raised by the novel field of immunometabolism to prevent inflammation in age-related disorders and repurposing anti-inflammatory drugs as a therapeutic option for treating several age-associated diseases, must be considered a priority.

## Figures and Tables

**Table 1:** Mechanism of action of CR mimetics.

CR Mimetic	Target	Mechanism of Action	Cell/Tissue/Organism	References
NAD^+^ precursors	Autophagy-mitochondria	Sirt1 activation	Fly, worm, mouse	[68,69,70,71,72,73,74,75,76]
	Inflammation	Pro-inflammatory cytokine levels decrease	Mouse	[66,78,79]
		TNF-α expression blockade	Macrophages	[81]
		Inflammasome inhibition	Mouse brain	[84]
		Microglia and neutrophil infiltration blockade	Microglia, neutrophils	[90]
		Inflammaging dampening	Aged humans	[93]
Resveratrol	Autophagy-mitochondria	AMPK and Sirt1 activation	Worm, mouse	[99,100]
	Inflammation	Pro-inflammatory cytokine levels decrease	Mouse, humans	[95,98]
		Anti-inflammatory T cell increase	T cells	[112]
		Adhesion molecule repression	Endothelial cells	[115,116,117,118]
		NF-κB inhibition	Macrophages, microglia	[111,121]
Metformin	Autophagy-mitochondria	AMPK and Sirt1 activation	Worm, mouse	[125]
	Mitochondria	mtROS production decrease	Mouse	[127]
	Inflammation	Adhesion molecule repression	Vascular endothelial cells	[129]
		SASP blockade	Senescent cells	[131]
		NF-κB and JNK inhibition	Mouse	[132]
		Memory T cell differentiation	Mouse	[136]
		Pro-inflammatory cytokine expression and immune cell infiltration blockade	Mouse brain	[137,146,147]
		Microglia activation inhibition	Mouse brain	[139]
		Intestinal inflammation reduction	Mouse intestine	[155,156]
Spermidine	Autophagy-mitochondria	Protein acetylation inhibition	Yeast, worm, fly	[157,158]
	Inflammation	Inflammaging dampening	Mouse	[159]
		Adhesion molecule repression	Lymphocytes	[162]
		CD8^+^ T cell response restoration	T cells	[164]
		Pro-inflammatory cytokine downregulation	Macrophages and human mononuclear cells	[165,166]
		Intestinal barrier homeostasis	Intestinal epithelial cells	[168]
		NF-κB inhibition	Microglia	[169]
		M2-type polarization	Macrophages	[171]
Rapamycin	Autophagy-mitochondria	mTOR inhibition	Fly, mouse, worm	[30,172,179]
	Inflammation	TNF-α expression blockade	Monocytes	[182,183]
		NK cell inhibition	NK cells	[184]
		PD1^+^ T cell blockade and memory CD8^+^ T cell increase	T cells	[186,187]
		SASP blockade and senescent cell elimination	Senescent cells	[188,189]
		Microglia activation inhibition	Mouse brain	[192,193,194,195,197]
		Immune cell infiltration blockade	γδ T cells, granulocytes	[197,198]
		Regulatory T cell polarization	Brain T cells	[197,198]
		Intestinal barrier homeostasis	Fly	[200]

The table summarizes the mechanisms by which CR mimetics exert their beneficial function and the tissues or organisms in which those effects have been validated.
